# Quantum information, cognition, and music

**DOI:** 10.3389/fpsyg.2015.01583

**Published:** 2015-10-21

**Authors:** Maria L. Dalla Chiara, Roberto Giuntini, Roberto Leporini, Eleonora Negri, Giuseppe Sergioli

**Affiliations:** ^1^Dipartimento di Lettere e Filosofia, Università di FirenzeFirenze, Italy; ^2^Dipartimento di Pedagogia, Psicologia, Filosofia, Università di CagliariCagliari, Italy; ^3^Dipartimento di Ingegneria Gestionale, dell'Informazione e della Produzione, Università di BergamoDalmine, Italy; ^4^Scuola di Musica di Fiesole, San Domenico di FiesoleFiesole, Italy

**Keywords:** Turing machines, quantum computation, quantum information, semantics, music cognition

## Abstract

Parallelism represents an essential aspect of human mind/brain activities. One can recognize some common features between psychological parallelism and the characteristic parallel structures that arise in quantum theory and in quantum computation. The article is devoted to a discussion of the following questions:

1. a comparison between classical probabilistic Turing machines and quantum Turing machines.
2. possible applications of the quantum computational semantics to cognitive problems.
3. parallelism in music.

## 1. Introduction

Parallelism represents an essential aspect of the activities of human brain and mind. One can recognize some common features between psychological parallelism and the characteristic parallel structures that arise in quantum theory and in quantum computation, being responsible for the extraordinary efficiency and speed of quantum computers.

Quantum parallelism and classical parallelism are deeply different, although it is sometimes claimed that quantum Turing machines are nothing but special examples of classical probabilistic Turing machines^1^. But what exactly are quantum Turing machines? So far, the literature has not provided a rigorous “institutional” concept of *quantum Turing machine*. Some definitions seem to be based on a kind of “imitation” of the classical definition of *Turing machine*, by referring to a *tape* (where the symbols are written) and to a *moving head* (which changes its position on the tape)^2^. These concepts, however, seem to be hardly applicable to physical quantum computers. Both in the classical and in the quantum case, it is expedient to consider a more abstract concept: the notion of *state machine*, which neglects both tapes and moving heads. Every finite computational task realized in different computational models proposed in the literature can be simulated by a state machine^3^. In order to compare classical and quantum parallelism, we will analyze the concepts of (classical) *deterministic state machine*, (classical) *probabilistic state machine*, and *quantum state machine*. On this basis we will discuss the question: to what extent can quantum state machines be simulated by probabilistic state machines? (Sections 2, 3).

In the investigation about possible links between *quantum structures* and *psychological structures* a useful tool is represented by a special form of quantum logical semantics (called *quantum computational semantics*) that has been inspired by the theory of quantum computation. We will see how this semantics can be naturally applied to a formal analysis of musical compositions, where *parallel structures, ambiguity, holism*, and *contextuality* play an essential role (Sections 4, 5)^4^.

Our analysis seems to confirm a general conjecture that has been defended and discussed in different research-fields: the basic concepts of the quantum-theoretic formalism (which had for a long time been regarded as mysterious and potentially paradoxical) seem to have a *universal* interest that goes beyond the domain of microphysical phenomena.

## 2. Classical deterministic and probabilistic machines

We will first introduce a formal definition for the notion of *deterministic state machine*. On this basis, *probabilistic state machines* will be represented as stochastic variants of deterministic machines, which are able to calculate different outputs with different probability-values.

**Definition 1**. *Deterministic state machine*.

A *deterministic state machine* is an abstract system **M** based on the following elements:

1. A finite set S of *internal states*, which contains an *initial state s*_*in*_ and includes a set of *halting states* S_*halt*_ = {*s*_*halt*_*j*__ | *j* ∈ *J*}.
2. A finite alphabet, which can be identified with the set {0, 1} of the two classical bits. Any *register* represented by a bit-sequence *w* = (*x*_1_, …, *x*_*n*_) is a *word* (of length *n*). Any pair (*s, w*) consisting of an internal state *s* and of a word *w* represents a possible *configuration* of **M**, which is interpreted as follows: **M** is in the internal state *s* and *w* is the word written on an ideal tape.
3. A set of words that represent possible *word-inputs* for **M**.
4. A *program*, which is identified with a finite sequence of *rules*:
  $$(R_{0},\ldots ,R_{t}).$$
  Each *R*_*i*_ is a partial function that transforms configurations into configurations. We may have: *R*_*i*_ = *R*_*j*_ with *i* ≠ *j*. The number *i*, corresponding to the rule *R*_*i*_, represents the *i*-th step of the program. The following conditions are required:
  4.1 The rule *R*_0_ is defined for any configuration (*s*_0_, *w*_0_), where *s*_0_ is the initial state *s*_*in*_ and *w*_0_ is a possible word-input. We have:
    $$R_{0}:(s_{0},w_{0})\mapsto (s_{1},w_{1}),$$
    where *s*_1_ is different from the initial state and from all halting states (if *t* ≠ 0).
  4.2 For any *i* (0 < *i* < *t*),
    $$R_{i}:(s_{i},w_{i})\mapsto (s_{i\;+\;1},w_{i\;+\;1}),$$
    where *s*_*i* + 1_ is different from all *s*_*i*_, …, *s*_0_ and from all halting states.
  4.3 *R*_*t*_: (*s*_*t*_, *w*_*t*_) ↦ (*s*_*t* + 1_, *w*_*t* + 1_),
    where *s*_*t* + 1_ is a halting state.
    Each configuration (*s*_*i* + 1_, *w*_*i* + 1_) represents the *output* for the step *i* and the *input* for the step *i* + 1.

The concept of *computation* of a deterministic state machine can be now defined as follows.

**Definition 2**. *Computation of a deterministic state machine*.

A *computation* of a deterministic state machine **M** is a finite sequence of configurations

$$((s_{0},w_{0}),\ldots ,(s_{t\;+\;1},w_{t\;+\;1})),$$

where:

1. *w*_0_ is a possible word-input of **M**.
2. *s*_0_, …, *s*_*t* + 1_ are different internal states of **M** such that: *s*_0_ = *s*_*in*_ and *s*_*t* + 1_ is a halting state.
3. For any *i* (0 ≤ *i* ≤ *t*),
  $$(s_{i+1},\;w_{i+1})\;=\;R_{j}((s_{i},\;w_{i})),$$
  where *R*_*i*_ is the *i*-th rule of the program.

The configurations (*s*_0_, *w*_0_) and (*s*_*t* + 1_, *w*_*t* + 1_) represent, respectively, the *input* and the *output* of the computation; while the words *w*_0_ and *w*_*t* + 1_ represent, respectively, the *word-input* and the *word-output* of the computation.

Apparently, each deterministic state machine is devoted to a single task that is determined by its program.

Let us now turn to the concept of *probabilistic state machine*. The only difference between deterministic and probabilistic state machines concerns the program, which may be stochastic in the case of a probabilistic state machine (**PM**). In such a case, instead of a sequence of rules, we will have a sequence (*Seq*_0_, …, *Seq*_*t*_) of sequences of rules such that:

$$\begin{array}{l} Seq_{0}=(R_{0_{1}},\ldots ,R_{0_{r}})\\ \;\ldots \ldots \ldots \\ Seq_{t}=(R_{t_{1}},\ldots ,R_{t_{l}}). \end{array}$$

Each rule *R_i_j__* (occurring in the sequence *Seq*_*i*_) is associated to a probability-value *p_i_j__* such that:

$$\sum_{j}p_{i_{j}}=1.$$

From an intuitive point of view, *p_i_j__* represents the probability that the rule *R_i_j__* be applied at the *i*-th step. A deterministic state machine is, of course, a special case of a probabilistic state machine characterized by the following property: each sequence *Seq*_*i*_ consists of a single rule *R*_*i*_.

Any probabilistic state machine naturally gives rise to a graph-structure for any choice of an input-configuration *conf*_0_ = (*s*_0_, *w*_0_). As an example, consider the following simple case: a probabilistic state machine **PM** whose program consists of two sequences, each consisting of two rules:

$$\begin{array}{l} Seq_{0}=(R_{0_{1}},R_{0_{2}})\\ Seq_{1}=(R_{1_{1}},R_{1_{2}}). \end{array}$$

**Figure 1:**
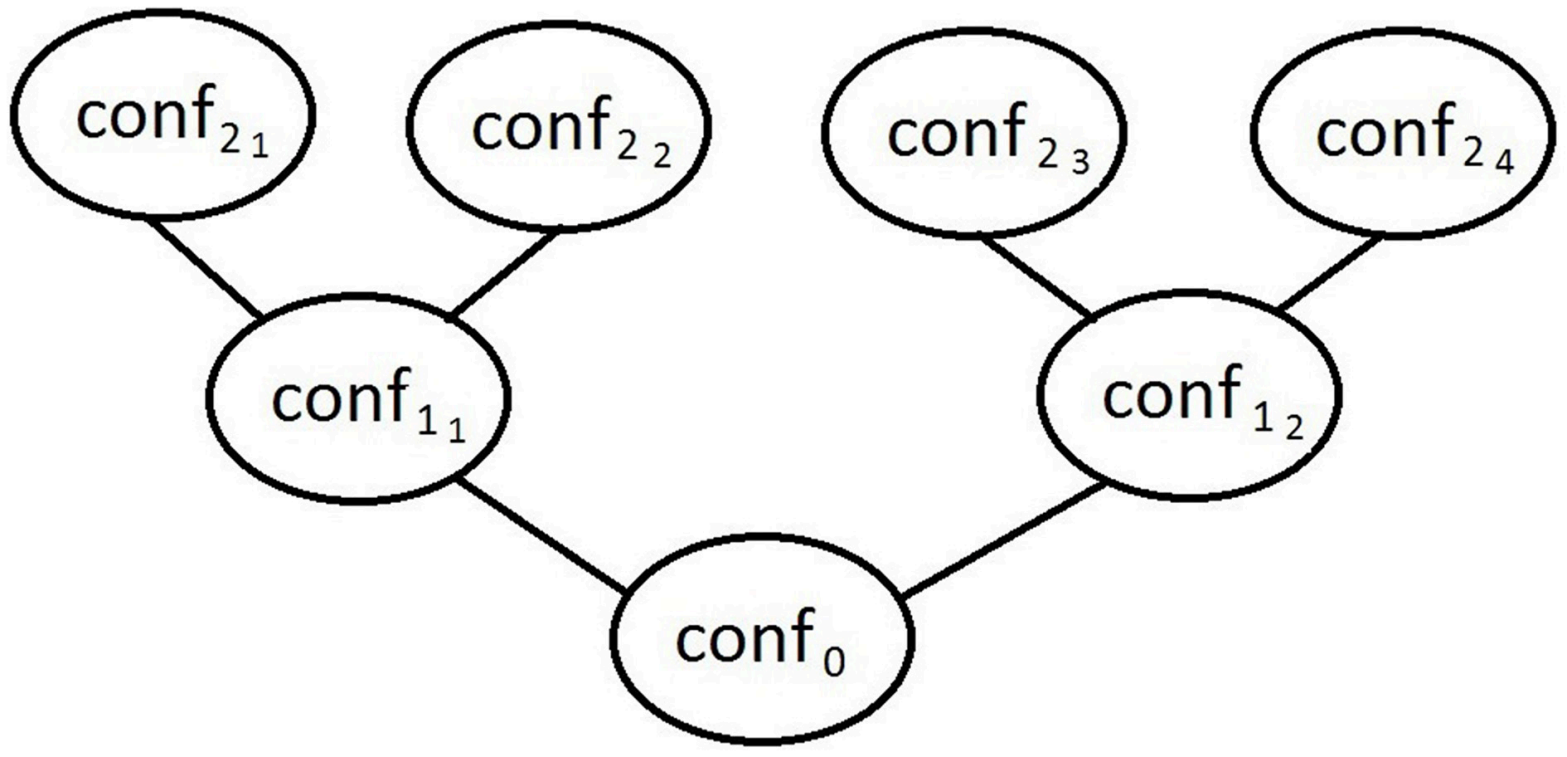
**The graph of PM**.

The graph associated to **PM** for the configuration *conf*_0_ is illustrated by Figure 1.

How do probabilistic machines compute? In order to define the concept of *computation* of a probabilistic machine, let us first introduce the notions of *program-path* and of *computation-path* of a given probabilistic machine.

**Definition 3**. *Program-path and computation-path*.

Let **PM** be a probabilistic state machine with program (*Seq*_0_, …, *Seq*_*t*_).

- A *program-path* of **PM** is a sequence
  $$P\;=\;(R_{0_{h}},\ldots ,R_{i_{j}},\ldots ,R_{t_{k}}),$$
  consisting of *t* rules, where each *R_i_j__* is a rule from *Seq*_*i*_ (probabilistically independent of all other rules of P).
- For any choice of an input (*s*_0_, *w*_0_), any program-path P determines a sequence of configurations
  $$\mathrm{CP}=((s_{0},w_{0}),\ldots ,(s_{i},w_{i}),\ldots ,(s_{t\;+\;1},w_{t\;+\;1})),$$
  where (*s*_*i* + 1_, *w*_*i* + 1_) = *R_i_j__*(*s*_*i*_, *w*_*i*_) and *R_i_j__* is the *i*-th element of P. This sequence is called the *computation-path* of **PM** determined by the program-path P and by the input (*s*_0_, *w*_0_). The configuration (*s*_*t* + 1_, *w*_*t* + 1_) represents the output of CP.

Any program-path P = (*R*_0_*h*__, …, *R_i_j__*, …, *R_t_k__*) has a well-determined probability-value *p*(P), which is defined as follows (in terms of the probability-values of its rules):

$$p(P):=p_{0_{h}}\cdot \ldots \cdot p_{i_{j}}\cdot \ldots \cdot p_{t_{k}}.$$

As expected, the probability-value of a program-path P naturally determines the probability-values of all corresponding computation-paths. It is sufficient to put:

p(CP):=p(P).

Consider now the set **P**_**PM**_ of all program-paths and the set **CP**_**PM**_ of all computation-paths of a probabilistic machine **PM**. One can easily show that:

$$\sum_{i}\{p(P_{i})|P_{i}\in P_{PM}\}=\sum_{i}\{p(CP_{i})|CP_{i}\in CP_{PM}\}=1.$$

On this basis the concept of *computation* of a probabilistic state machine can be defined as follows.

**Definition 4**. *Computation of a probabilistic state machine*.

A *computation* of a probabilistic state machine **PM** with input (*s*_0_, *w*_0_) is the system of all computation-paths of **PM** with input (*s*_0_, *w*_0_).

Unlike the case of deterministic state machines, a computation of a probabilistic state machine does not yield a unique output. For any choice of a configuration-input (*s*_0_, *w*_0_), the computation-output is a system of possible configuration-outputs $\left(s_{t+1}^{i},w_{t+1}^{i}\right)$, where each $\left(s_{t+1}^{i},w_{t+1}^{i}\right)$ corresponds to a computation-path CP_*i*_. As expected, each $\left(s_{t+1}^{i},w_{t+1}^{i}\right)$ has a well-determined probability-value that is defined as follows:

$$\begin{array}{l} p((s_{t+1}^{i},w_{t+1}^{i})):=\sum_{i}\{p(CP_{i})|\text{the configuration-output of}\\ \;CP_{i}\text{ is }\left(s_{t+1}^{i},w_{t+1}^{i}\right)\}. \end{array}$$

One can easily show that the sum of the probability-values of all configuration-outputs of any machine **PM** is 1.

## 3. Quantum state machines

The strong parallelism that characterizes quantum computers is based on two quantum-theoretic notions that have been often described as mysterious and potentially paradoxical: *superposition* and *entanglement*. For the readers who are not expert of quantum theory it is expedient to recall some concepts of the quantum formalism that are used in quantum computation^5^. The basic idea is that any *piece of quantum information* is mathematically represented as a possible *state* of a quantum system that can store and transmit the information in question. In the simplest situations one is dealing with a single particle *S* (say, an electron or a photon), whose “mathematical environment” is a special example of a vector space: the two-dimensional Hilbert space ℂ^2^, based on the set of all ordered pairs of complex numbers. The canonical (orthonormal) basis of ℂ^2^ consists of the two following unit-vectors:

|0〉=(1,0);  |1〉=(0,1),

which represent, in this framework, the two classical bits (0 and 1), or (equivalently) the two classical truth-values (*Falsity* and *Truth*). A *pure state* corresponds to a *maximal piece of information* that cannot be consistently extended to a richer knowledge. Such state is represented as a unit-vector |ψ〉 that can be expressed as a *superposition* of the two elements of the canonical basis of ℂ^2^:

$$|\psi \rangle =c_{0}|0\rangle +c_{1}|1\rangle ,$$

where *c*_0_ and *c*_1_ (also called *amplitudes*) are complex numbers such that $|c_{0}{|}^{2}+|c_{1}{|}^{2}=1$.

The physical interpretation of |ψ〉 (also called *qubit-state* or, briefly, *qubit*) is the following: the physical system *S* in state |ψ〉 might satisfy the physical properties that are *certain* for the bit |0〉 with probability $|c_{0}{|}^{2}$ and might satisfy the physical properties that are *certain* for the bit |1〉 with probability $|c_{1}{|}^{2}$. Due to the characteristic indeterminism of quantum theory, the pure state |ψ〉 is at the same time a *maximal and logically incomplete piece of information* that cannot *decide* some important physical properties of the system *S*. Accordingly, from an intuitive point of view, one can say that |ψ〉 describes a kind of *cloud of potential properties* that might become *actual* when a measurement is performed. Measuring a physical quantity (by means of an apparatus associated to the canonical basis) determines a sudden transformation of the qubit |ψ〉 either into the bit |0〉 or into the bit |1〉. Such transformation is usually called *collapse of the wave-function*.

Not all states associated to a physical system *S* are pure. Non-maximal pieces of information can be represented as *mixtures of pure states* (special examples of operators called *density operators*). In the space ℂ^2^ a density operator ρ can be represented as a convenient finite sum of projection-operators:

$$\rho =\sum_{i}w_{i}P_{|\psi_{i}\rangle },$$

where *w*_*i*_ are real numbers such that $\underset{i}{\sum }w_{i}=1$, while each *P*_|_ψ__*i*_〉_ is a projection-operator that projects along the direction of |ψ〉. Notice that such representation is not generally unique. A density operator that cannot be represented as a projection *P*_|ψ〉_ is called a *proper mixture*. While pure states codify an essential indetermination of some relevant properties of the quantum system under investigation, mixtures may correspond to an *epistemic uncertainty* of the observer. Unlike pure states (which always satisfy some well-determined properties), there are mixtures that cannot decide any (non-trivial) property of the associated system. An example of this kind is the state $\rho =\frac{1}{2}\text{I}$, where I is the identity operator of the space ℂ^2^.

As happens in classical information theory, quantum computation also needs complex pieces of information, which are supposed to be stored by composite quantum systems (generally consisting of *n* subsystems). Accordingly, one can naturally adopt the quantum-theoretic formalism for the mathematical representation of composite physical systems, based on the use of *tensor products* (special examples of products)^6^. While a single qubit is a unit-vector of the space ℂ^2^, a pure state representing a complex piece of information can be identified with a unit-vector of the *n*-fold tensor product of ℂ^2^:

$${⊗}^{n}{\mathbb{C}}^{2}=\underset{n-times}{\underset{︸}{{\mathbb{C}}^{2}⊗\ldots ⊗{\mathbb{C}}^{2}}}(\text{with }n\geq 1).$$

Such vectors are called *quregisters*. The canonical basis of the space ⊗^*n*^ℂ^2^ consists af all *registers*, products of bits that have the following form:

$$|x_{1}\rangle ⊗\ldots ⊗|x_{n}\rangle \text{ (where any }x_{i}\text{ is either }0\text{ or }1).$$

Instead of |*x*_1_〉 ⊗ … ⊗ |*x*_*n*_〉, it is customary to write |*x*_1_, …, *x*_*n*_〉. Any quregister can be represented as a superposition of registers:

$$|\psi \rangle =\sum_{i}c_{i}|x_{i_{1}},\ldots ,x_{i_{n}}\rangle ,$$

where *c*_*i*_ are complex numbers such that $\underset{i}{\sum }|c_{i}{|}^{2}=1.$

A tensor product |ψ_1_〉 ⊗ … ⊗ |ψ_*n*_〉 (of *n* quregisters) is often briefly indicated by: |ψ_1_〉…|ψ_*n*_〉.

Quantum computation makes essential use of some characteristic quantum states that are called *entangled*. In order to illustrate the concept of entanglement from an intuitive point of view, let us refer to a simple paradigmatic case. We are concerned with a composite physical system *S* consisting of two subsystems *S*_1_ and *S*_2_ (say, a two-electron system). By the quantum-theoretic rules that concern the mathematical description of composite systems, all states of *S* shall live in the tensor product H = H_1_ ⊗ H_2_, where H_1_ and H_2_ are the Hilbert spaces associated to the systems *S*_1_ and *S*_2_, respectively. The observer has a *maximal information* about *S*: a *pure state* |ψ〉 of H. What can be said about the states of the two subsystems? Due to the form of |ψ〉, such states cannot be pure: they are represented by two identical *mixtures*, which codify a “maximal degree of uncertainty.” A typical possible form of |ψ〉 is the following *Bell-state*:

$$|\psi \rangle =\frac{1}{\sqrt{2}}(|0,0\rangle +|1,1\rangle ),$$

which lives in the space ℂ^2^ ⊗ ℂ^2^, whose canonical basis consists of the four vectors |0, 0〉, |0, 1〉, |1, 0〉, |1, 1〉.

This gives rise to the following physical interpretation: the global system *S* might satisfy the properties that are *certain* either for the state |0, 0〉 or for the state |1, 1〉 with probability-value $\frac{1}{2}$. At the same time, |ψ〉 determines that the *reduced state* of both subsystems (*S*_1_ and *S*_2_) is the mixture $\frac{1}{2}\text{I}$. Although it is not determined whether the state of the global system *S* is |0, 0〉 or |1, 1〉, the two subsystems *S*_1_ and *S*_2_ can be described as “entangled,” because in both possible cases they would satisfy the same properties, turning out to be *indistinguishable*. As a consequence, any measurement performed by an observer either on system *S*_1_ or on system *S*_2_ would instantaneously transform the *potential* properties of both subsystems into *actual* properties (by *collapse of the wave-function*).

The celebrated “Einstein–Podolsky–Rosen paradox”(*EPR*) is based on a similar physical situation. As is well-known, what mainly worried Einstein was the possibility of “non-local effects:” the subjective decision of an observer (who may choose among different *incompatible observables* to be measured on the system *S*_1_) seems to determine the instantaneous emergence of an actual property for the system *S*_2_, which might be very “far” from *S*_1_ (possibly inaccessible by means of a light-signal). Interestingly enough, in the framework of quantum computation, entangled states have been often used as a powerful resource, even from a technological point of view (for instance, in the applications to teleportation-phenomena and to quantum cryptography).

As expected, quantum computation cannot be identified with a “static” representation of pieces of information. What is important is the dynamic *process* of information that gives rise to quantum computations. Such process is mathematically performed by *quantum logical gates* (briefly, *gates*): special examples of *unitary operators* that transform quregisters into quregisters in a reversible way. Since in quantum theory the time-evolution of all physical systems is mathematically described by unitary operators, one can say that quantum computations can be regarded as the time-evolution of some special quantum objects.

We will now introduce the definition of *quantum state machine*, which represents a quantum counterpart of the classical notion of *deterministic state machine*. From an intuitive point of view, any quantum state machine can be regarded as a kind of quantum superposition of many classical deterministic state machines. Some definitions of *quantum Turing machine* discussed in the literature are based on a strong idealization: no limit is assumed for the length of the registers occurring in a computation. This corresponds to the classical assumption according to which a Turing machine is equipped with an infinite tape. We will consider a more realistic concept, closer to physical quantum computers, which are of course always bound to a limited memory.

**Definition 5**. *Quantum state machine*.

A *quantum state machine* is an abstract system **QM** associated to a (finite-dimensional) Hilbert space H^**QM**^ whose unit-vectors |ψ〉 represent possible pure states of a quantum system that could physically implement the computations of the state machine. The space H^**QM**^ has the following form:

$$H^{QM}=H^{H}⊗H^{S}⊗H^{W}.$$

The following conditions are required:

1. H^*H*^ (which represents the halting-space) is the space ℂ^2^, where the two elements of the canonical basis ({|0〉_*H*_, |1〉_*H*_}) correspond to the states “the machine does not halt” and “the machine halts,” respectively.
2. H^S^ (which represents the internal-state space) is associated to a finite set S of classical internal states. We require that H^S^ = ⊗^*m*^ℂ^2^, where 2^*m*^ is the cardinal number of S. Accordingly, the set S can be one-to-one associated to a basis of H^S^.
3. H^*W*^ (which represents the word-space) is identified with a Hilbert space ⊗^*n*^ℂ^2^ (for a given *n* ≥ 1). The number *n* determines the length of the registers |*x*_1_, …, *x*_*n*_〉 that may occur in a computation. Shorter registers |*x*_1_, …, *x*_*h*_〉 (with *h* < *n*) can be represented in the space ⊗^*n*^ℂ^2^ by means of convenient *ancillary bits*.
  Let *B*^**QM**^ be a basis of H^**QM**^, whose elements are unit-vectors having the following form:
  $$|\varphi_{i}\rangle =|h_{i}\rangle |s_{i}\rangle |x_{i_{1}},\ldots ,x_{i_{n}}\rangle ,$$
  where |*h*_*i*_〉 belongs to the basis of H^*H*^, while |*s*_*i*_〉 belongs to the basis of H^*S*^.
  Any unit-vector |ψ〉 of H^**QM**^ that is a superposition of basis-elements |φ_*i*_〉 represents a possible *computational state* of **QM**. The expected interpretation of a computational state
  $$|\psi \rangle =\sum_{i}c_{i}|h_{i}\rangle |s_{i}\rangle |x_{i_{1}},\ldots ,x_{i_{n}}\rangle$$
  is the following:
  - the machine in state |ψ〉 might halt with probability $|c_{i}{|}^{2}$ (if |*h*_*i*_〉 = |1_*H*_〉) or with probability $1-|c_{i}{|}^{2}$ (if |*h*_*i*_〉 = |0_*H*_〉).
  - the machine in state |ψ〉 might correspond to the classical configuration (*s*_*i*_, (*x*_*i*_1__, …, *x*_*i*_*n*__)) with probability $|c_{i}{|}^{2}$.
    Hence, the state |ψ〉 describes a kind of *quantum co-existence* of different classical deterministic configurations.
4. The set of *possible inputs* of **QM** is identified with the set of all computational states that have the following form:
  $$|\psi \rangle =\sum_{i}|0_{H}\rangle |s_{in}\rangle |x_{i_{1}},\ldots ,x_{i_{n}}\rangle .$$
5. Like a deterministic state machine, a quantum state machine **QM** is characterized by a *program*. In the quantum case, a program is identified with a sequence of unitary operators of H^**QM**^:
  $$(U_{0},\ldots ,U_{t}),$$
  where we may have: *U*_*i*_ = *U*_*j*_ with *i* ≠ *j*.
  The following conditions are required:
  - for any possible input |ψ_0_〉, *U*_0_(|ψ_0_〉) = |ψ_1_〉 is a superposition of basis-elements having the following form:
    $$|h_{i}^{1}\rangle |s_{i}^{1}\rangle |x_{i_{1}}^{1},\ldots ,x_{i_{n}}^{1}\rangle ,$$
    where all $s_{i}^{1}$ are different from *s*_*in*_ and $\text{|}h_{i}^{1}\rangle =\;|0_{H}\rangle$, if *t* ≠ 0.
  - For any *j* (0 < *j* < *t*), *U*_*j*_(|ψ_*j*_〉) = |ψ_*j* + 1_〉 is a superposition of basis-elements having the following form:
    $$|0_{H}\rangle |s_{i}^{j+1}\rangle |x_{i_{1}}^{j+1},\ldots ,x_{i_{n}}^{j+1}\rangle .$$
  - *U*_*t*_(|ψ_*t*_〉) = |ψ_*t* + 1_〉 is a finite superposition of basis-elements having the following form:
    $$|1_{H}\rangle |s_{halt_{j}}\rangle |x_{i_{1}}^{t+1},\ldots ,x_{i_{n}}^{t+1}\rangle .$$

The concept of *computation* of a quantum state machine can be now defined in a natural way.

**Definition 6**. *Computation of a quantum state machine*.

Let **QM** be a quantum state machine, whose program is the operator-sequence (*U*_0_, …, *U*_*t*_) and let |ψ_0_〉 be a possible input of **QM**. A *computation* of **QM** with input |ψ_0_〉 is a sequence of computational states of **QM**

$$QC=(|\psi_{0}\rangle ,\ldots ,|\psi_{t+1}\rangle ),$$

such that: |ψ_*i* + 1_〉 = *U*_*i*_(|ψ_*i*_〉), for any *i* (0 ≤ *i* ≤ *t*).

The vector |ψ_*t* + 1_〉 represents the output of the computation, while the density operator $Red^{3}(\text{|}\psi_{t\;+\;1}\rangle )$ (the reduced state of |ψ_*t* + 1_〉 with respect to the third subsystem) represents the word-output of the computation.

Like all abstract notions of *quantum computer*, the concept of *quantum state machine* gives rise to some critical questions that have been often discussed in the literature. Two important problems (which cannot have any counterpart in the case of classical computation) are the following:

- How shall we interpret the operation of “reading the output” of a computation of a given machine? What is the role of the *collapse of the wave-function* during a reading-action?
- Is it possible to measure the halting state without disturbing the configuration-state?

Consider now a quantum state machine whose program is

$$(U_{0},\ldots ,U_{t}).$$

Each *U*_*i*_ naturally determines a corresponding word-operator $U_{i}^{W}$, defined on the word-space H^*W*^. Generally, it is not guaranteed that all word-operators are unitary. But it is convenient to refer to quantum state machines that satisfy this condition. In this way, any quantum state machine (whose word-space is ⊗^*n*^ℂ^2^) determines a *quantum circuit*, consisting of a sequence of unitary operators (*gates*):

$$(U_{0}^{W},\ldots ,U_{t}^{W}),$$

where *n* represents the *width*, while *t* + 1 represents the *depth* of the circuit.

**Figure 2:**
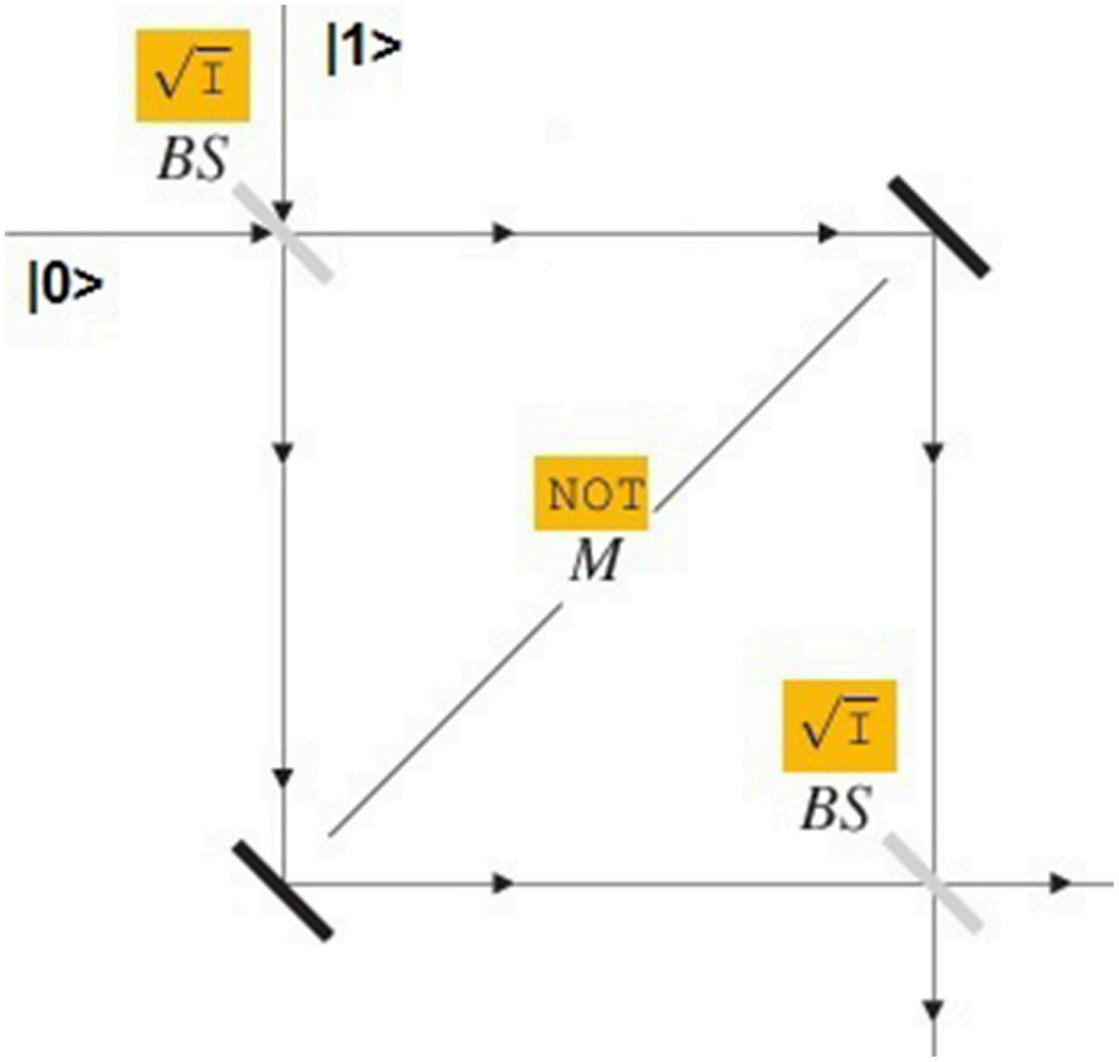
**The Mach–Zehnder interferometer**.

To what extent can quantum state machines be simulated by classical probabilistic state machines? In order to discuss this important question, let us refer to a celebrated quantum experiment, based on the *Mach–Zehnder interferometer* (represented by Figure 2).

The physical situation can be sketched as follows. Consider a photon-beam (possibly consisting of a single photon) and assume that |0〉 describes the state of photons moving along the *x* direction, while |1〉 describes the state of photons moving along the *y* direction. All photons go through a first *beam splitter* that “splits” them giving rise to the following effect: within the box each photon follows a path corresponding either to the *x*-direction or to the *y*-direction with probability $\frac{1}{2}$. Soon after, on both paths, all photons are reflected by a *mirror* that inverts their direction. Finally, the photons pass through a second beam splitter that determines the output-state. Suppose that all photons entering into the interferometer-box are moving in the *x*-direction. According to a “classical way of thinking” we would expect that the photons detected at the end of the process will move either along the *x*-direction or along the *y*-direction with probability $\frac{1}{2}$. The result of the experiment is, instead, completely different: the Mach–Zehnder interferometer *always* transforms the input-state |0〉 into the output-state |0〉; while the input-state |1〉 is transformed into |1〉.

From a mathematical point of view, such a “surprising” result can be explained by using, in an essential way, the concept of superposition. The apparatuses (used in the Mach–Zehnder experiment) can be mathematically represented by two important gates. A beam splitter can be regarded as a physical implementation of the *Hadamard-gate* $\sqrt{\text{I}}$ (also called *square root of identity*), which is defined as follows (on the canonical basis of ℂ^2^):

$$\sqrt{I}|0\rangle =\frac{1}{\sqrt{2}}(|0\rangle +|1\rangle );\sqrt{I}|1\rangle =\frac{1}{\sqrt{2}}(|0\rangle -|1\rangle ).$$

**Figure 3:**
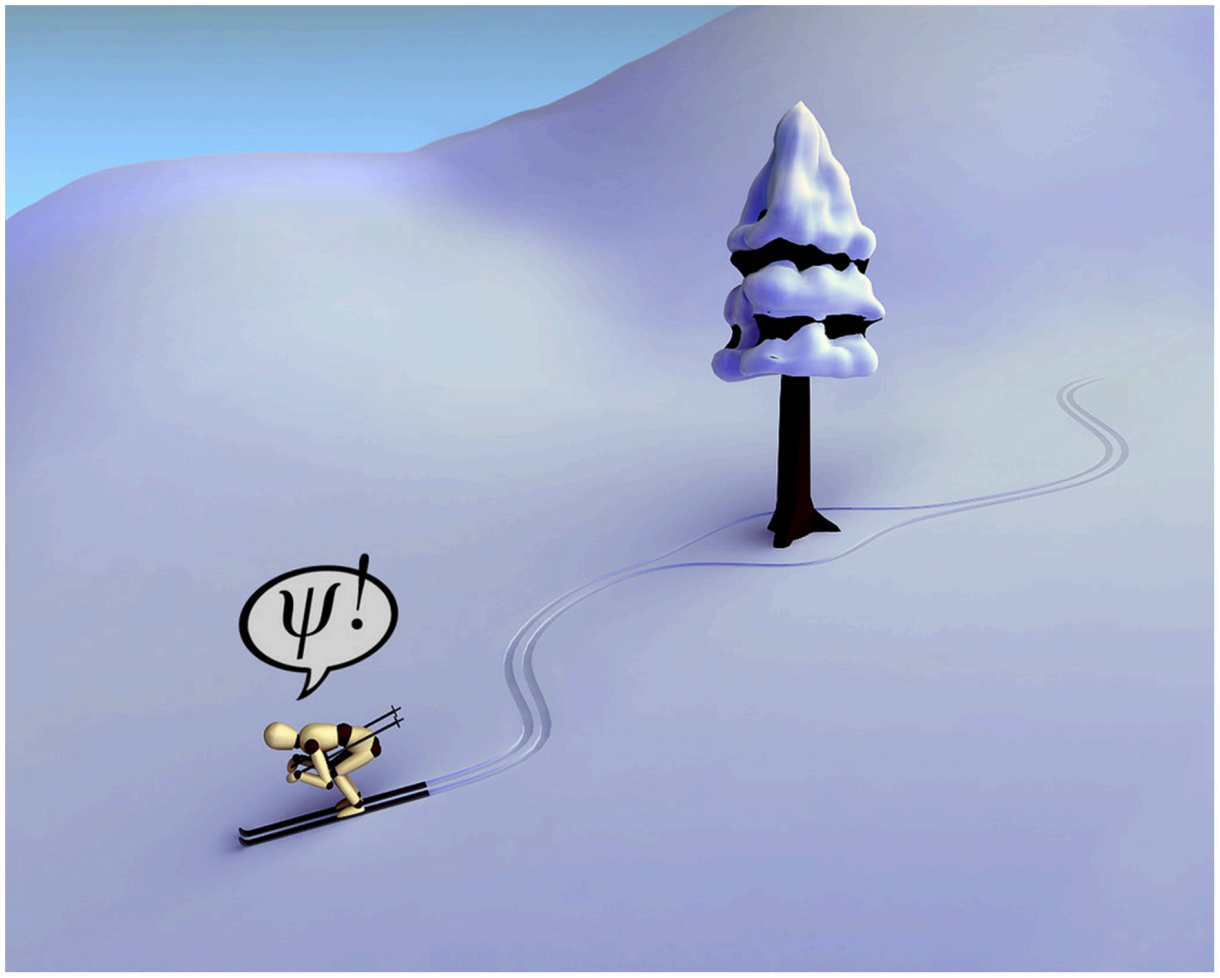
**The quantum skier**.

Apparently, the Hadamard-gate transforms the two classical bits |0〉 and |1〉 into two (different) genuine superpositions. As a consequence, within the Mach–Zehnder box a photon in state $\frac{1}{\sqrt{2}}(|0\rangle +|1\rangle )$ turns out to satisfy at the same time two alternative properties: the property of moving along the *x*-direction and the property of moving along the *y*-direction. We have here a characteristic quantum parallelism: a single photon “goes along” two different paths at the same time! Metaphorically, situations of this kind have been sometimes compared to the puzzling behavior of a “quantum skier” who runs at the same time on the left and on the right side of a given tree (see Figure 3).

The second apparatus of the Mach–Zehnder interferometer (the mirror), can be regarded as a physical implementation of another important gate, the *negation* NOT (a quantum generalization of the classical negation), which is defined as follows:

NOT|0〉=|1〉;NOT|1〉=|0〉.

Accordingly, the *Mach–Zehnder circuit* can be identified with the following sequence of three gates (all defined on the space ℂ^2^):

$$(\sqrt{I},NOT,\sqrt{I}).$$

Let us now apply the Mach–Zehnder circuit to the input |0〉. We obtain:

$\sqrt{\text{I}}:\text{ |}0\rangle \;\mapsto \;\frac{1}{\sqrt{2}}(\text{|}0\rangle +\text{ |}1\rangle );\text{NOT }:\;\frac{1}{\sqrt{2}}(\text{|}0\rangle +\text{ |}1\rangle )\;\mapsto \;\frac{1}{\sqrt{2}}(\text{|}0\rangle +\text{ |}1\rangle );\;\sqrt{\text{I}}:\;\frac{1}{\sqrt{2}}(\text{|}0\rangle +\text{ |}1\rangle )\mapsto \text{ |}0\rangle$.

We can see, in this way, how the Mach–Zehnder circuit transforms the input-state |0〉 into the output-state |0〉. In a similar way, the input-state |1〉 is transformed into the output-state |1〉.

Is there any natural “classical counterpart” for the Hadamard-gate? A natural candidate might be a particular example of a probabilistic state machine that we can conventionally call *the classical probabilistic NOT-state machine* (**PM**^NOT^). Such machine can be defined as follows:

- The set of possible word-inputs of **PM**^NOT^ is the set of words {(0), (1)}.
- The program of **PM**^NOT^ consists of the following sequence of rules:
  $$Seq_{0}=(R_{0_{1}},R_{0_{2}}),$$

where:

*R*_0_1__ : (*s*_*in*_, (*x*)) ↦ (*s*_*halt*_*j*__, (*x*)) and $p\left(R_{0_{1}}\right)=\frac{1}{2}$;

*R*_0_2__ : (*s*_*in*_, (*x*)) ↦ (*s*_*halt*_*j*__, (1 − *x*)) and $p\left(R_{0_{2}}\right)=\frac{1}{2}$.

Consider, for instance, the input (*s*_*in*_, (0)). The output will be the following set:

$$\left\{\left(s_{halt_{j}},(0)),(s_{halt_{j}},(1)\right)\right\}.$$

**Figure 4:**
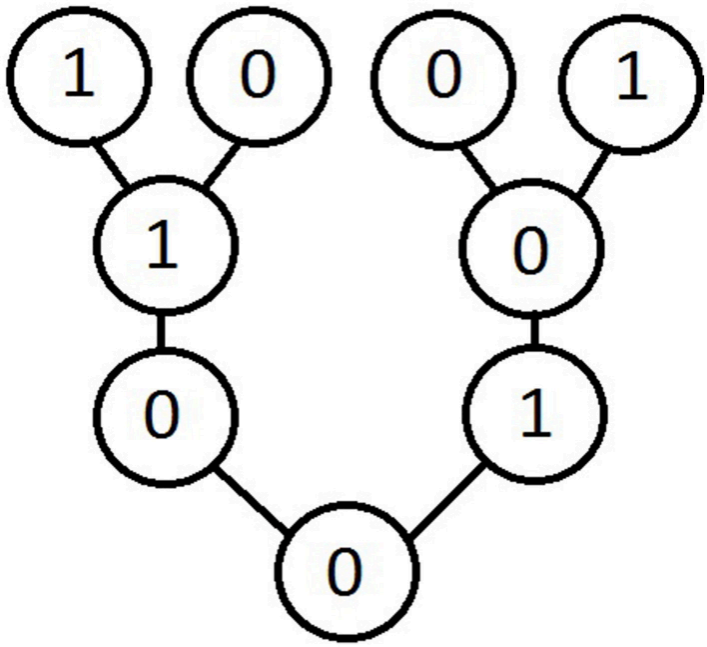
**A word-graph for a “classical probabilistic Mach–Zehnder state machine”**.

On this basis, a “classical probabilistic Mach–Zehnder state machine” would determine (for the word-input (0)) the word-graph illustrated by Figure 4.

Such a machine turns out to compute both the words (0) and (1) with probability $\frac{1}{2}$. Interestingly enough, this is the same probabilistic result that is obtained in the quantum case, when one performs a measurement inside the interferometer-box. In such a case, photons behave like “normal skiers,” who pass *either* at the right *or* at the left side of a tree (where *or* represents here, of course, the exclusive disjunction).

The arguments we have developed seem to confirm the following conjecture: the characteristic superposition-patterns, that may occur during a quantum computation (when no measurement is performed during the computation-process), cannot be generally represented by probabilistic state machines. Quantum parallelism (based on superpositions) and classical parallelism are deeply different.

## 4. Quantum parallelism, psychological parallelism, and quantum computational semantics

What kind of similarity can be recognized between quantum parallel structures and different forms of psychological parallelism? Trying to represent the human mind as a kind of system of quantum state machines would be, of course, naive and misleading. In spite of many important results in the framework of neurosciences, the complex network that connects human conscious and unconscious thoughts is still quite mysterious^7^. Quantum-like superpositions can be reasonably applied to represent some aspects of such complex networks. Even quantum *interference phenomena* (with the characteristic constructive and destructive effects) can find some natural psychological interpretations.

According to an interesting hypothesis (discussed by the neuroscientist Boncinelli, 2012), the mysterious emergence of an *act of consciousness* can be represented as a sudden transition from a parallel structure to a linear one. Is it reasonable to conjecture that such transition could be described as a kind of “psychological collapse of the wave-function?”

In the investigations about possible links between *quantum structures* and *psychological structures* a useful tool is represented by a special form of quantum logical semantics (called *quantum computational semantics*) that has been naturally inspired by the theory of quantum computation^8^.

Let us briefly recall the basic ideas of this semantics. We can refer a first-order language L, whose non-logical alphabet contains individual terms (variables and names), predicates and sentential constants. Interpreting the language L means associating to any formula α a *meaning*, identified with a piece of quantum information that can be stored by a quantum system. Accordingly, any possible meaning of α is represented by a possible (pure or mixed) state of a quantum system: generally, a density operator ρ_α_ that lives in a Hilbert space H^α^, whose dimension depends on the linguistic complexity of α.

The logical operators of L are associated to special examples of Hilbert-space operations that have a characteristic dynamic behavior, representing possible computation-actions. The logical connectives are interpreted as particular (reversible) gates, like the negation NOT, the Hadamard-gate $\sqrt{\text{I}}$, the Toffoli-gate T (which allows us to define a reversible conjunction AND). At the same time, the logical quantifiers (∀, ∃) are interpreted as possibly irreversible quantum operations. Since the universe of discourse (which the language refers to) may be indeterminate, the use of quantum quantifiers may give rise to a reversibility-breaking, which is quite similar to what happens in the case of measurement-phenomena.

Due to the characteristic features of quantum holism, meanings turn out to behave in a *holistic* and *contextual* way: the density operator ρ_α_ (which represents the *global* meaning of a formula α) determines the *contextual meanings* of all parts of α (which can be obtained by applying the *reduced-state function* to ρ_α_). As a consequence, it may happen that the meaning of a formula is an entangled pure state, while the meanings of its parts are proper mixtures. In such cases, the meaning of a global expression turns out to be more precise than the meanings of its parts. It is also admitted that one and the same formula receives different contextual meanings in different contexts.

As an example, consider the atomic sentence “Alice is pretty” (formalized as **Pa**). In order to store the information expressed by this sentence, we need three quantum objects whose states represent the pieces of information corresponding, respectively, to the predicate **P**, to the name **a** and to the truth-degree according to which the individual denoted by the name **a** satisfies the property denoted by the predicate **P**. Accordingly, the meaning of the sentence **Pa** can be identified with a (pure or mixed) state ρ_**Pa**_ living in the tensor-product space H^**Pa**^ = ⊗^3^ℂ^2^. In order to obtain the contextual meanings of the linguistic parts of **Pa** it is sufficient to consider the two reduced states *Red*^1^(ρ_**Pa**_) and *Red*^2^(ρ_**Pa**_), which describe (respectively) the states of the first and of the second subsystem of the quantum object that stores the information expressed by the sentence **Pa**. From a logical point of view, *Red*^1^(ρ_**Pa**_) and *Red*^2^(ρ_**Pa**_) can be regarded as two *intensional meanings*: a property-concept and an individual concept, respectively; while ρ_**Pa**_ represents a *propositional concept* (or *event*).

Like formulas, sequences of formulas also can be interpreted according to the quantum computational rules. As expected, a possible meaning of the sequence (α_1_, …, α_*n*_) will be a density operator ρ_(_α__1_, …, α_*n*_)_ living in a Hilbert space H^(α_1_,…,α_*n*_)^, whose dimension depends on the linguistic complexity of the formulas α_1_, …, α_*n*_.

In this framework one can develop an abstract theory of *vague possible worlds*. Consider a pair

$$W=((\alpha_{1},\ldots ,\alpha_{n}),\rho_{\left(\alpha_{1},\ldots ,\alpha_{n}\right)}),$$

consisting of a sequence of formulas and of a density operator that represents a possible meaning for our sequence. It seems reasonable to assume that *W* describes a *vague possible world*, a kind of *abstract scene* where most events are characterized by a “cloud of ambiguities,” due to quantum uncertainties. In some cases *W* might be exemplified as a “real” scene of a theatrical play or as a vague situation that is described either in a novel or in a poem. And it is needless to recall how ambiguities play an essential role in literary works.

As an example, consider the following vague possible world:

$$W=((Pab),\rho_{\left(Pab\right)}),$$

where **Pab** is supposed to formalize the sentence “Alice is kissing Bob,” while ρ_**Pab**_ corresponds to the pure state

$$|\Psi_{Pab}\rangle =|\varphi \rangle ⊗\frac{1}{\sqrt{2}}(|0,1\rangle )+|1,0\rangle )⊗|1\rangle ,$$

where |φ〉 lives in the space ℂ^2^, while |Ψ〉_**Pab**_ lives in the space ⊗^4^ℂ^2^. Here the reduced state of |Ψ〉_**Pab**_ that describes the pair (Alice, Bob) has the typical form of an entangled state; consequently, the states describing the two individuals Alice and Bob are two identical mixed states. In the context |Ψ〉_**Pab**_ Alice and Bob turn out to be indistinguishable: it is not determined “who is who” and “who is kissing whom.” It is not difficult to imagine some “real” theatrical scenes representing ambiguous situations of this kind.

## 5. A quantum semantics for music

An abstract version of the quantum computational semantics can be applied to a formal analysis of musical compositions, where both *musical ideas* and *extra-musical meanings* are generally characterized by some essentially vague and ambiguous features^9^.

Any musical composition (say, a sonata, a symphony, an opera,…) is, generally, determined by three elements:

- a *score*;
- a set of *performances*;
- a set of musical *thoughts* (or *ideas*), which represent possible *meanings* for the *musical phrases* written in the score.

While scores represent the syntactical component of musical compositions, performances are physical events that occur in space and time. From a logical point of view, we could say that performances are, in a sense, similar to *extensional meanings*, i.e., well-determined systems of objects which the linguistic expressions refer to.

Musical thoughts (or ideas) represent, instead, a more mysterious element. Is it reasonable to assume the existence of such ideal objects that are, in a sense, similar to the *intensional meanings* investigated by logic? Is there any danger to adhere, in this way, to a form of *Platonism*? When discussing semantic questions, one should not be “afraid” of Platonism. In the particular case of music, a composition cannot be simply reduced to a score and to a system of sound-events. Between a score (which is a system of signs) and the sound-events created by a performance there is something intermediate, represented by the musical ideas that underlie the different performances. This is the abstract environment where normally live both composers and conductors, who are accustomed to study scores without any help of a material instrument.

Following the rules of the quantum semantics, *musical ideas* can be naturally represented as superpositions that ambiguously describe a variety of co-existent thoughts. Accordingly, we can write:

$$|\mu \rangle =\sum_{i}c_{i}|\mu_{i}\rangle ,$$

where:

- |μ〉 is an abstract object representing a musical idea that *alludes* to other ideas |μ_*i*_〉 (possible *variants* of |μ〉 that are, in a sense, all co-existent);
- the number *c*_*i*_ measures the “weigth” of the component |μ_*i*_〉 in the context |μ〉.

As happens in the case of composite quantum systems, musical ideas (which represent possible meanings of *musical phrases* written in a score) have an essential *holistic* behavior: the meaning of a global musical phrase determines the *contextual meanings* of all its parts (and not the other way around).

An important feature of music is the capacity of *evoking* extra-musical meanings: subjective feelings, situations that are vaguely imagined by the composer or by the interpreter or by the listener, real or virtual theatrical scenes (which play an essential role in the case of lyric operas and of *Lieder*). The interplay between musical ideas and extra-musical meanings can be naturally represented in the framework of our quantum semantics, where extra-musical meanings can be dealt with as special examples of vague possible worlds.

We can refer to the abstract tensor product of two spaces

MSpace⊗WSpace,

where:

- *MSpace* represents the space of musical ideas |μ〉.
- *WSpace* represents the space of vague possible worlds, dealt with as special examples of abstract objects |*w*〉 that can be evoked by musical ideas.

Following the quantum-theoretic formalism, we can distinguish between *factorized* and *non-factorized* global musical ideas. A factorized global musical idea will have the form:

|M〉=|μ〉⊗|w〉.

But we might also meet entangled global musical ideas, having the form:

$$|M\rangle =c_{1}(|\mu_{1}\rangle ⊗|w_{1}\rangle )+c_{2}(|\mu_{2}\rangle ⊗|w_{2}\rangle ).$$

As is well-known, music gives rise to a special kind of psychological experience, where some complex parallel structures are consciously grasped, in a way that may appear miraculous. Paradigmatic examples arise, for instance, in the case of trios or quartets of lyric operas. In such cases, the listener perceives a *global polyphonic structure*; at the same time, he/she is able to follow (at least to a certain extent) the different melodic lines and even the different thoughts and feelings of the characters who are singing. As an example, it may be interesting to consider three great masterpieces of the history of lyric operas: the quartet of Act 1 in Beethoven's *Fidelio*, the quartet of Act 3 in Verdi's *Rigoletto* and the trio of Act 3 of *Der Rosenkavalier* by Richard Strauss. The parallel structures that arise in these three examples have some significant differences both from the musical and from the semantic point of view.

**Figure 5:**
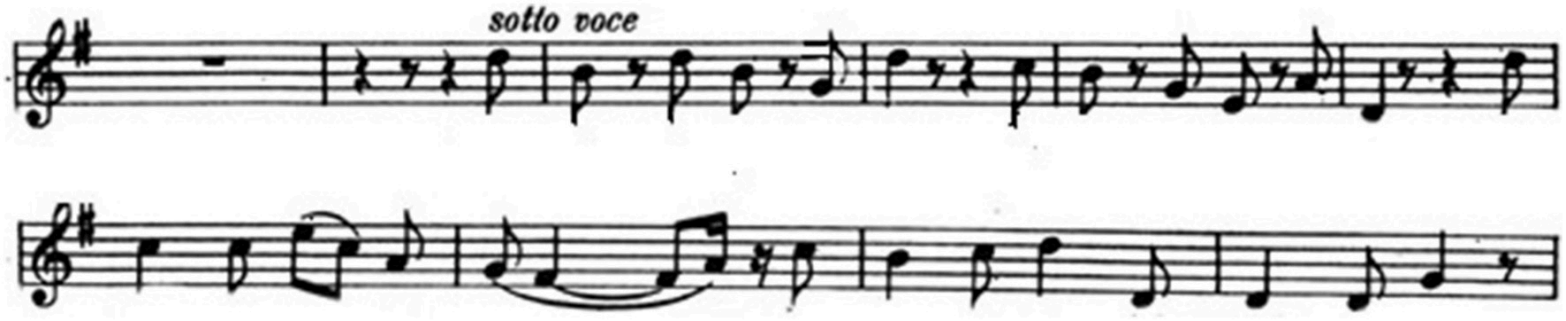
**The quartet-theme**.

In *Fidelio*'s quartet the psychological contraposition between the four characters (Marzelline, Leonore, Rocco, Jaquino) is realized by means of a single musical theme that is successively sung by the four singers (Figure 5).

It is amazing how Beethoven succeeds in expressing, by one and the same theme, different attitudes and emotions: the joyful hope of Marzelline, the doubts and the anguish of Leonore, the paternal satisfaction of Rocco, the jealous rage of Jaquino. The whole context is dominated by strong ambiguities and antagonistic elements: the contrast between an improbable family-portrait and the cruel jail-environment, the contradictions of Rocco (who is at the same time a fond father and an accomplice of the prison-system), the sexual ambiguity of Leonore, the loving heroin who has disguised herself as a man (Fidelio), in the attempt to save her husband, the prisoner Florestan. The musical result is an extraordinary and highly emotional polyphonic construction based on very simple musical components.

**Figure 6:**
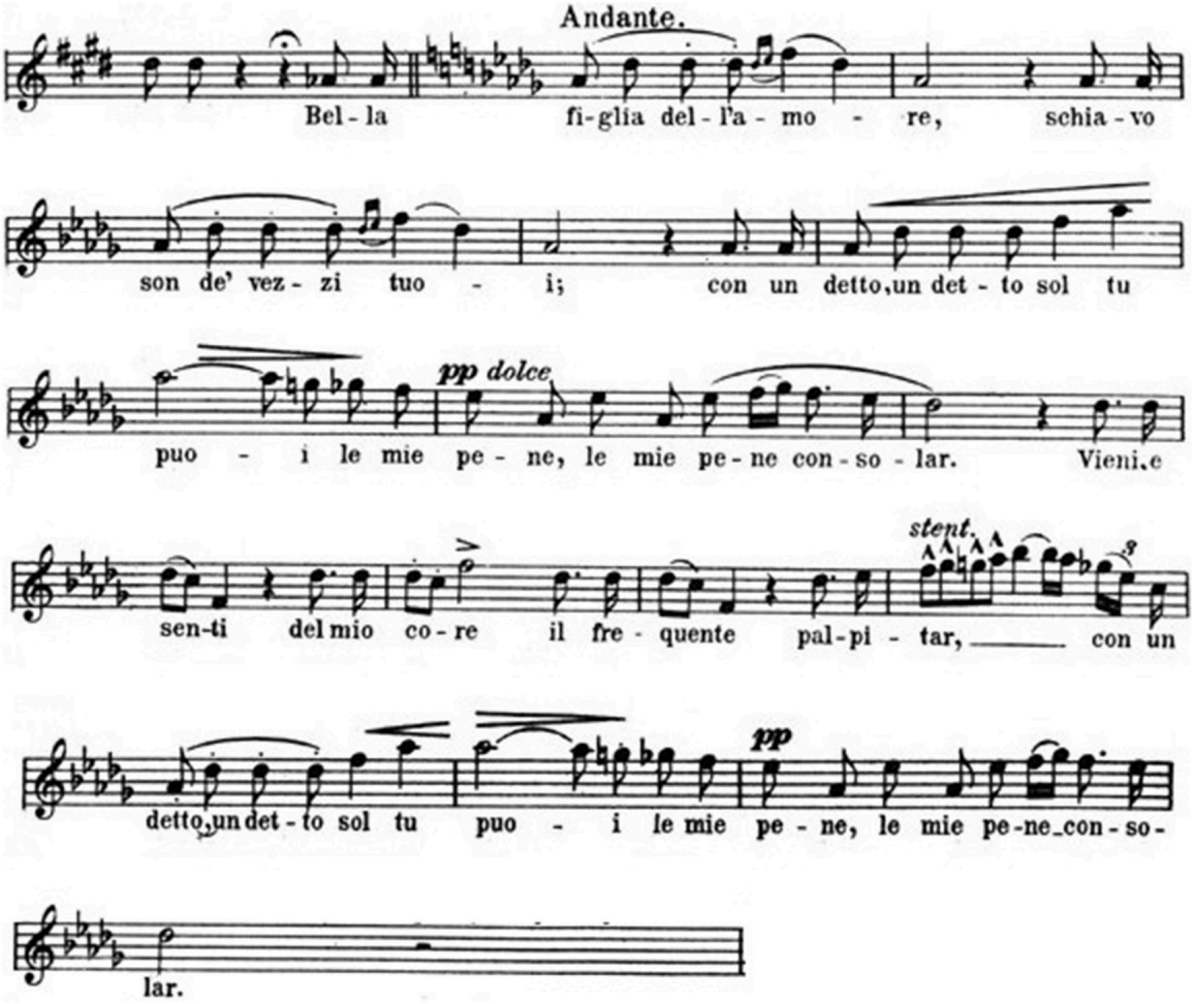
**The Duke**.

The structure of *Rigoletto*'s quartet is completely different. All characters are associated to specific musical themes that are repeated with some variations. The leading musical idea is represented by the wonderful theme sung by the Duke of Mantova at the very beginning (Figure 6)^10^.

**Figure 7:**
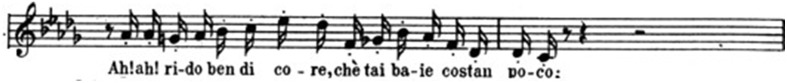
**Maddalena**.

Like Mozart's *Don Giovanni*, Verdi's Duke is a cynic seducer, who may appear sweet and sincere with his victims. And music often exalts a paradoxical co-existence of contradictory psychological attitudes. All contrasts are emphasized in the quartet by the sordid environment, where a crime is going to be committed. Maddalena's answer to the Duke is based on a fully different theme, a *staccato*-sequence of sixteenth-notes (Figure 7)^11^.

**Figure 8:**
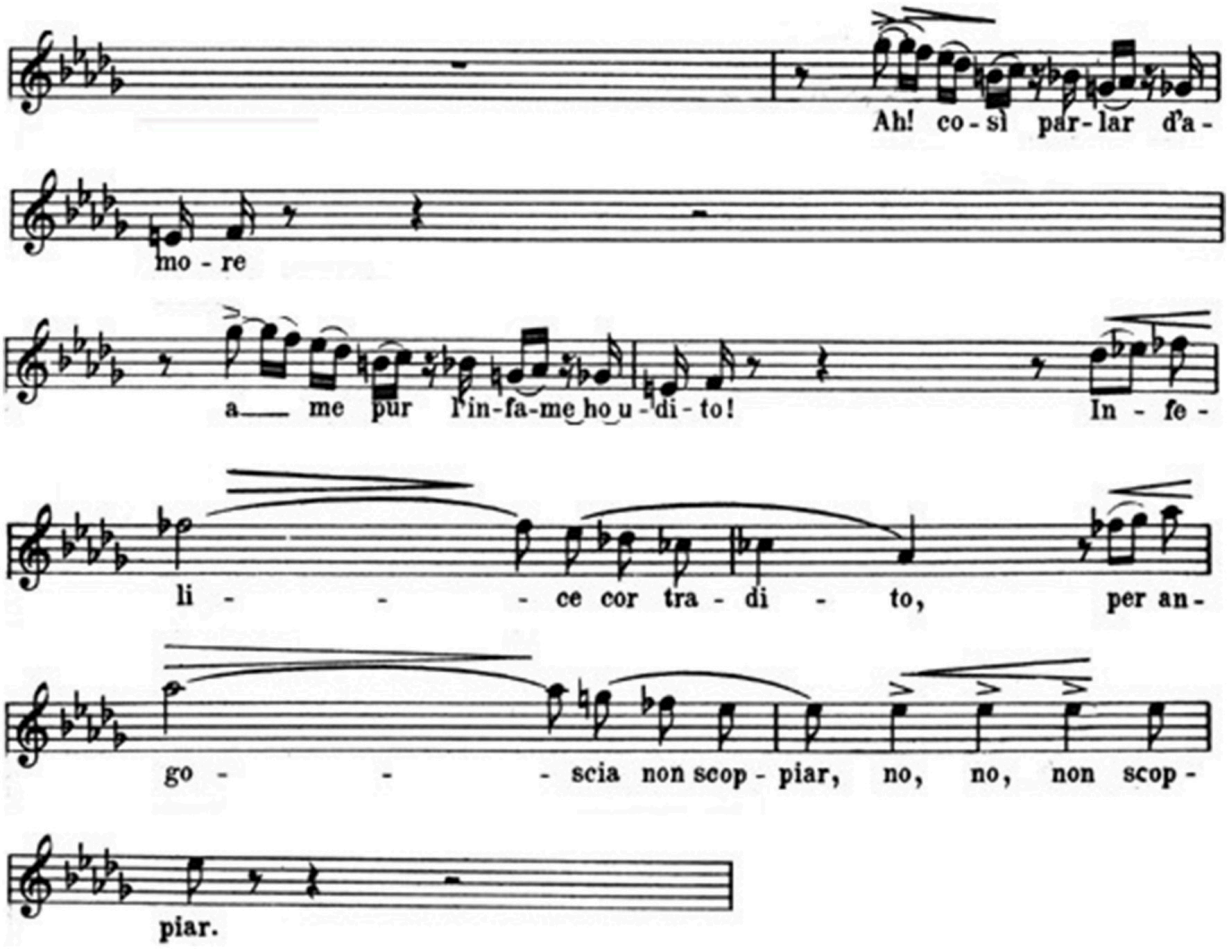
**Gilda**.

Both the music and the text reflect Maddalena's ambiguity: she is a prostitute who is playing a traditional seductive role; at the same time she is also instrumental to a murder-project. Gilda's entrance (soon after Maddalena's first phrase) determines a sudden dramatic change. What Gilda sings is a cry of sorrow, interrupted by some short pauses and *appoggiaturas* that seem to describe desperate sobs (Figure 8)^12^.

One has often discussed the reasons that may have led Gilda to her unreasonable sacrifice for an unworthy man who had deceived her. Representing Gilda as a naive and modest girl is, however, misleading and in contrast with the greatness expressed by the music. Gilda's death-choice can be perhaps better understood as a suicide, caused by an unendurable disillusion. Rigoletto's role in the quartet is musically less “visible.” His mind is completely absorbed in the vengeance-project (“la vendetta”) that shall be shortly accomplished. From a musical point of view, the quartet is constructed as a polyphonic structure, where the four voices are interlaced, each preserving its own musical, semantic and psychological autonomy.

*Der Rosenkavalier* by Strauss belongs to a musical and literary world that is somewhat far both from *Fidelio* and from *Rigoletto*. Different forms of ambiguity are exalted in this opera, which is characterized by an extraordinary unity of music and text, written by the great poet Hugo von Hofmannsthal. The theme of sexual ambiguity is here developed by the character of Octavian, the *Rosenkavalier* whose role is sung by a mezzo-soprano. Although Octavian may recall Mozart's Cherubino, ambiguities are in Strauss' opera more sophisticated: in two different situations Octavian disguises himself as a woman in order to make fun of the rude fiancé of the fascinating girl Sophie. Interestingly enough, some interpreters of the role of Octavian have told how often they have been puzzled by their “oscillating identity” during the opera's performance.

A different and deeper “identity-question” is evoked in a splendid aria sung by the *Marschallin* in Act 1. After a passionate night spent with her lover Octavian, the lady is troubled by some sad thoughts about the flowing of time and the mysterious co-existence of different identities of one and the same person in different stages of life. She sings:

*Aber wie kann das wirklich sein*,

*dass ich die kleine Resi war*,

und dass ich einmal die alte Frau sein werd'

.......................................

Wie kann denn das geschehen?

Wie macht denn das der liebe Gott?

*Wo ich doch immer die gleiche bin*.

*Und wenn er's schon so machen muss*,

warum lasst er mich denn zuschaun dabei

mit gar so klarem Sinn?

Warum versteckt er's nicht vor mir?

*Das alles ist geheim, so viel geheim* ^13^.

One is dealing with an extraordinary poetic and musical representation of a “hard” scientific and philosophical problem, that modern philosophers of science usually call “the genidentity-question”^14^.

**Figure 9:**
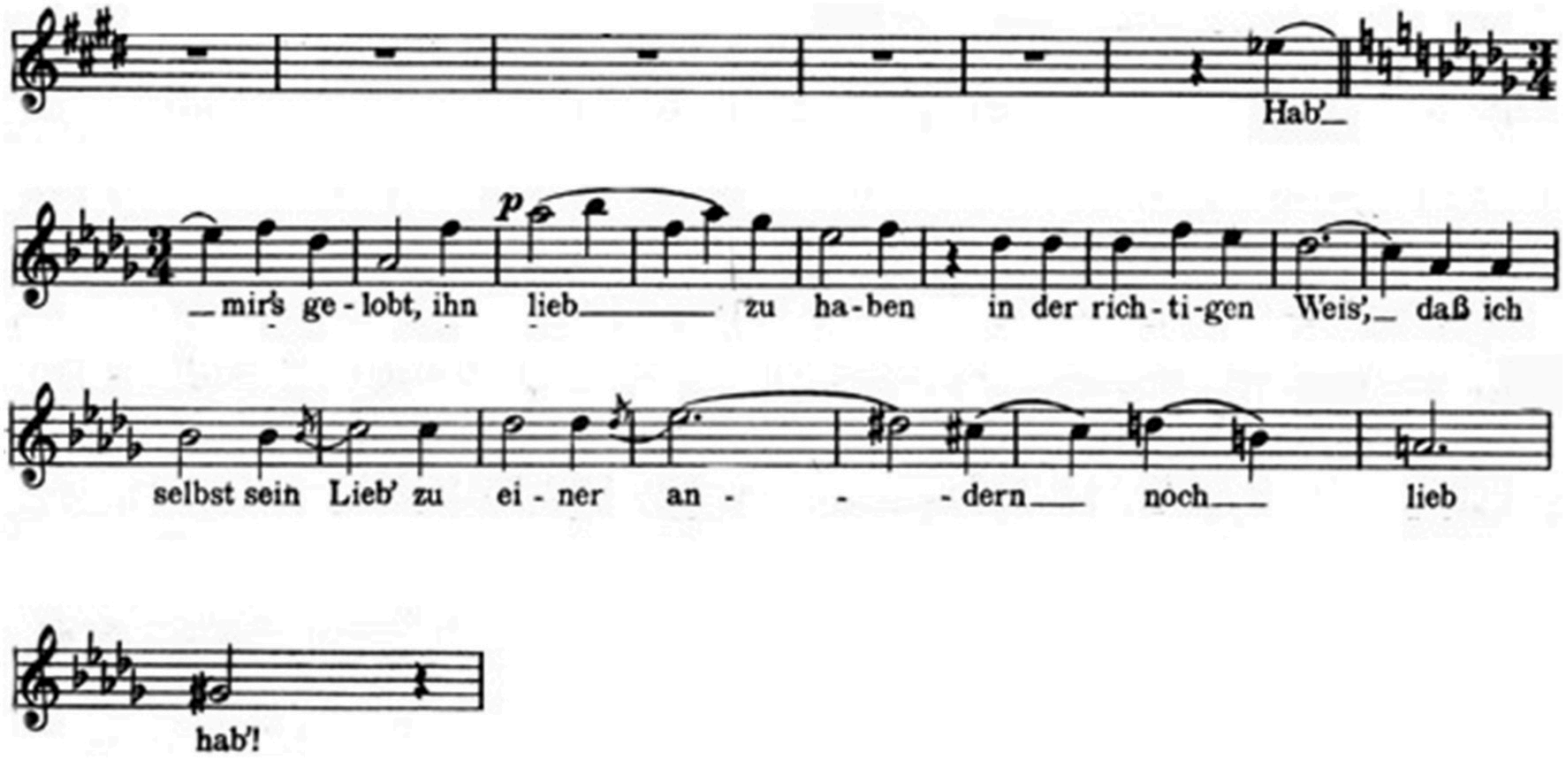
**The Marschallin**.

The trio performed at the end of the opera by three female voices (the Marschallin, Sophie, Octavian) is a wonderful polyphonic construction, where the three characters express different thoughts and feelings, which are not generally associated to some specific musical themes (unlike the case of *Rigoletto*'s quartet). The main theme is sung at the very beginning by the Marschallin (Figure 9)^15^.

By this deeply moving musical phrase the Marschallin expresses her extreme act of love, which is to renounce love. Her choice might recall what Violetta Valery sings in Verdi's *La Traviata:*

*Dite alla giovine sì bella e pura*^16^

although Violetta and the Marschallin are, of course, completely different characters.

Sophie's entrance in the trio is, in a sense, surprising. She joins in, in the final part of the Marschallin's first phrase, just upon the critical word “andern” (“other”). Her intervention creates a sudden brief dissonance (a minor-second chord), which immediately disappears when the two womem (who are both in love with Octavian) harmonically conclude the phrase at a distance of a minor-third. What Sophie perceives is a strange religious atmosphere that she cannot really understand, since she is not aware of the *liason* between Octavian and the Marschallin. The *incipit* of the main theme (the characteristic imprinting of the whole trio) is then immediately transposed to a different key (from *D* flat major to *A* major) by Octavian, whose initial attitude seems to be mainly dominated by embarassing doubts and questions. But finally the reasons of love prevail over all doubts. At the end of the trio, while the two young lovers sing an expected “dich habe ich lieb” (“I love you”), the Marschallin concludes with an enigmatic phrase:

*als wie halt Männer das glücklich sein verstehen*^17^.

singing the last note alone over a perfect tonic chord.

The three examples of polyphonic constructions, created by Beethoven, Verdi, and Strauss, are all characterized by strong unitary conceptions, based on complex parallel networks of harmonic, melodic, timbric, and semantic relationships (which have been extensively analyzed in musicological literature^18^). At the same time, one can easily recognize some significant differences that distinguish the three cases, both from the musical and from the semantic point of view. The structure of Fidelio's quartet is very close to a *canon-form*, where the entrance of each voice is associated to a specific semantic connotation. Rigoletto's quartet is, instead, dominated by strong musical contrasts that reflect the conflicting feelings of four human beings, living in a highly dramatic situation. Finally, Strauss' trio seems to propose a kind of musical and semantic “peaceful resolution.” The trio is perceived by the listener as a strongly unitary musical idea that evolves in time. The three female voices are in a sense “entangled,” sometimes creating the illusion that a single voice is singing (as happens in the case of some entangled quantum objects, whose parts are indistinguishable). Such musical situations can be naturally represented in the framework of the quantum musical semantics, where *musical thoughts* are dealt with as holistic ideal objects that vaguely allude to a (possibly infinite) variety of co-existing ideas.

The analysis proposed in this article has concerned questions that belong to worlds apparently “far apart”: the theory of quantum computers, psychology, logical semantics, and music. A common pattern that arises in all these fields is a frequent and sometimes essential emergence of some characteristic parallel structures. We have seen how the quantum-theoretic concepts of *superposition* and *entanglement* have inspired the development of a “bridge-theory” (based on the quantum computational semantics) that can be usefully applied to a formal representation of different kinds of phenomena where parallelism plays a relevant role.

### Conflict of interest statement

The authors declare that the research was conducted in the absence of any commercial or financial relationships that could be construed as a potential conflict of interest.

## Footnotes

^1^See, for instance, Penrose (1994).

^2^See, for instance, Fouché et al. (2007).

^3^See, for instance, Savage (1998) and Gudder (1999).

^4^Some basic intuitive ideas of the quantum computational semantics are close to the “quantum cognition approach” that has been extensively developed in recent times (see, for instance, Aerts and Gabora, 2005a,b; Aerts and Sozzo, 2014). In both theories *concepts* and *thoughts* are represented as special abstract entities that can be described in the framework of the quantum-theoretic formalism. The technical developments of the two approaches are, however, different.

^5^A survey of quantum computation theory can be found, for instance, in Nielsen and Chuang (2000).

^6^The basic property of the tensor product H_1_ ⊗ H_2_ of two (finite-dimensional) Hilbert spaces H_1_ and H_2_ is the following: H_1_ ⊗ H_2_ is a Hilbert space that properly includes an isomorphic image of the Cartesian product H_1_ × H_2_ (consisting of all ordered pairs of vectors that belong to the spaces H_1_ and H_2_, respectively). Furthermore, H_1_ ⊗ H_2_ contains all possible superpositions of its elements. A vector |ψ〉 of H_1_ ⊗ H_2_ is called *factorized* iff |ψ〉 corresponds to a pair (|ψ_1_〉, |ψ_2_〉)∈H_1_ × H_2_. In such a case, it is customary to write: |ψ〉 = |ψ_1_〉 ⊗ |ψ_2_〉. Of course, not all vectors of H_1_ ⊗ H_2_ are factorized.

^7^As is well-known, the literature devoted to the study of parallel structures in the mind/brain-behavior is very rich. As an example, one can refer to some important contributions of Damasio (see, for instance, Damasio, 1999).

^8^See (Dalla Chiara et al., 2005, 2010, in press).

^9^See (Dalla Chiara et al., 2012).

^10^*Fairest daughter of love, I am a slave of your charms; with but a single word you could relieve my every pain. Come touch my breast and feel how my heart is racing. With but a single word you could relieve my every pain*.

^11^*Ah! Ah! That really makes me laugh, talk like that is cheap enough*.

^12^*Ah, these are the loving words the scoundrel spoke once to me! O wretched heart betrayed, do not break of sorrow*.

^13^*But how can it be that I was the little Resi and that I shall be the old lady. .… How can it come to pass? How can God decree it so? While, in fact, I am always the same. And if indeed it must be so, why does he let me look at it so clearly? Why does he not hide it to me? All this is a mystery, a great mystery*.

^14^The term “genidentity,” which refers to the problematic *identity of individuals through time* has been introduced by Lewin (in his doctoral thesis in 1922) and has been further investigated by Reichenbach and many other scholars. See, for instance, Reichenbach (1928).

^15^*I promised to love him in the right way, even to love his love for another woman*.

^16^*Tell the beautiful and pure girl*.

^17^*as far as men can understand happiness*.

^18^See, for instance, Budden (1983), Solomon (1998), Principe (2004).
